# Cloning, Expression, and Purification of Nucleoside Diphosphate Kinase from *Acinetobacter baumannii*

**DOI:** 10.1155/2013/597028

**Published:** 2013-04-11

**Authors:** Juhi Sikarwar, Sanket Kaushik, Mau Sinha, Punit Kaur, Sujata Sharma, Tej P. Singh

**Affiliations:** Department of Biophysics, All India Institute of Medical Sciences, New Delhi 110029, India

## Abstract

*Acinetobacter baumannii* is a multidrug resistant pathogenic bacteria associated with hospital acquired infections. This bacterium possesses a variety of resistance mechanisms which makes it more difficult to control the bacterium with conventional drugs, and, so far no effective drug treatment is available against it. Nucleoside diphosphate kinase is an important enzyme, which maintains the total nucleotide triphosphate pool inside the cell by the transfer of **γ**-phosphate from NTPs to NDPs. The role of nucleoside diphosphate kinase (Ndk) has also been observed in pathogenesis in other organisms. However, intensive studies are needed to decipher its other putative roles in *Acinetobacter baumannii*. In the present study, we have successfully cloned the gene encoding Ndk and achieved overexpression in bacterial host BL-21 (DE3). The overexpressed protein is further purified by nickel-nitrilotriacetic acid (Ni-NTA) chromatography.

## 1. Introduction 

*Acinetobacter baumannii* is an aerobic gram-negative bacterium, widely known for its multidrug resistance [1]. It is an opportunistic bacterial pathogen and possesses wide variety of resistance mechanisms [2–4]. It has high survival rate on abiotic surfaces and has the ability to persist in the hospital environment [3]. Hence, it is largely responsible for hospital-acquired infections as well as community-acquired infections [2, 3]. Previously, it has been reported that the infections caused by this pathogen were of moderate severity; however, the recently increased prevalence of this bacterial infection with serious consequences has been observed [3–5]. These include bacteremia, pneumonia, meningitis, urinary tract, and wound infections [4]. 

 Nucleoside diphosphate kinase (Ndk) is involved in the catalysis of the transfer of *γ*-phosphate from nucleoside triphosphate to nucleoside diphosphate and thus maintains the nucleotide pool [6, 7]. The dNTPs that are formed are used as precursors for DNA and RNA synthesis. NTPs, specifically GTP, are important for cellular macromolecular synthesis and signalling mechanisms. Ndk has been found to play a pivotal role in crucial events like bacterial growth, signal transduction, and pathogenicity [8]. The role of Ndk in regulating growth, formation of NTP, and cell surface polysaccharides has been seen in pathogens like *Pseudomonas aeruginosa *and* Mycobacterium tuberculosis* [8–10]. Mycobacterial Ndk has been found to inhibit phagosome maturation and helps in the survival of the bacterium within the macrophage [11]. Ndk has also been shown to form different oligomeric structures in different species which is often linked to its diversified functions [12–16]. However, the exact molecular mechanism of the structural and functional aspects of Ndk in *Acinetobacter baumannii* has not been fully deciphered. Here we report the cloning, expression, and purification of Ndk from *Acinetobacter baumannii*.

## 2. Materials and Methods

### 2.1. Gene Cloning

*Escherichia coli *strains DH5*α* (Invitrogen, California, USA) and BL-21 (DE3) (Novagen, Wisconsin, USA) were used for cloning and expression of the recombinant protein, respectively. The *E. coli* cells harbouring recombinant plasmids were grown aerobically at 37°C in Luria-Bertani (Merck, Darmstadt, Germany) broth with or without 50 *μ*g/mL kanamycin (Sigma, Saint Louis, MO, USA). Plasmid pET-28a (Novagen, Wisconsin, USA) was used as an expression vector. 

 The full coding sequence of Ndk was amplified by polymerase chain reaction (PCR) using forward primer (5′-GGA ATT CCA TAT GGC AAT TGA ACG TAC TTT G-3′) incorporated an *NdeI* restriction enzyme site and reverse primer (5′CCG CTC GAG TTA ACG AGT GCG TGG GCA -3′) incorporated an *XhoI* site. 

 The purified Ndk fragment was digested with the restriction enzymes *NdeI* and *XhoI* and ligated into the *NdeI*-*XhoI* sites of pET28a vector, which provides six His residues at the N-terminus of the expressed protein. Recombinant vector pET28a-Ndk was transformed into competent *E. coli* DH5*α* cells. The integrity of the recovered plasmid was confirmed by restriction endonuclease digestion and sequencing of the Ndk.

### 2.2. Expression and Purification

 For expression of the recombinant protein, pET28a-Ndk plasmid was transformed into competent Bl-21 (DE3) cells. Bl-21 (DE3) cells harbouring a pET28a-Ndk vector were grown in LB medium supplemented with kanamycin (50 *μ*g/mL) at 37°C with shaking (250 rpm) until the absorbance at 600 nm reached approximately 0.5. Then, isopropyl-*β*-D-thiogalactopyranoside (IPTG) was added to a final concentration of 0.4 mmol/L. The cells were incubated for a further 4 hours before being harvested. Pellet of Bl-21 cells harbouring Ndk was suspended by gentle stirring in buffer (50 mM, Tris 150 mM NaCl, pH 8). The suspension was subjected to sonication (five cycles, 1 minute each, with intervals of 1 min in ice) and then centrifuged for 20 minutes at 6000 ×g. After centrifugation, the supernatant and precipitate were examined by sodium dodecyl sulfate polyacrylamide gel electrophoresis (SDS-PAGE) to verify the position of the expressed protein. 

 Ndk was purified by affinity chromatography on a nickel-nitrilotriacetic acid (Ni-NTA) gel matrix (Qiagen, Crawley, United Kingdom). A column containing 3 mL of Ni-NTA resin was equilibrated with 10 volumes of buffer containing 50 mM Tris, 150 mM NaCl, (pH 8.0), and supernatant was loaded onto the column. The column was washed with 5 volumes of wash buffer containing 50 mM Tris pH 8.0, 150 mM NaCl, and 30 mM imidazole. The protein was then eluted by increasing the imidazole concentration to 300 mM. Fractions containing the recombinant protein were pooled and dialyzed to remove Imidazole. Protein concentrations were determined by Nanodrop analyzer (Bio-Rad, Hercules, CA, USA), and the purity was determined by SDS-PAGE and Coomassie blue staining. 

### 2.3. Characterization Studies of Ndk

 Fluorescence spectroscopy was used to further characterize the enzyme in terms of stability, pH, and substrate concentrations. The change in fluorescence emission spectra were recorded in F-7000 FL spectrophotometer at 25°C. The emission spectra were recorded in range of 300 nm–450 nm with excitation at 280 nm. The final concentration of the protein was 1.7 × 10^-5 ^M. Stability of the protein was measured by incubating the protein in increasing concentrations of urea from 0 M to 8 M overnight at 4°C and fluorescence spectra were recorded at each of the different concentrations of urea. The substrate binding characteristics of the enzyme were studied by titrating ATP at different concentrations from 2 × 10^−5 ^M–10 × 10^−5 ^M and spectra were recorded at each of these different ATP concentrations. For pH characterization, the change in spectra due to ATP binding was recorded by varying the pH of the buffer: acidic pH 5.6, neutral pH 7 and basic pH 8.5. 

## 3. Results and Discussion

**Figure 1:**
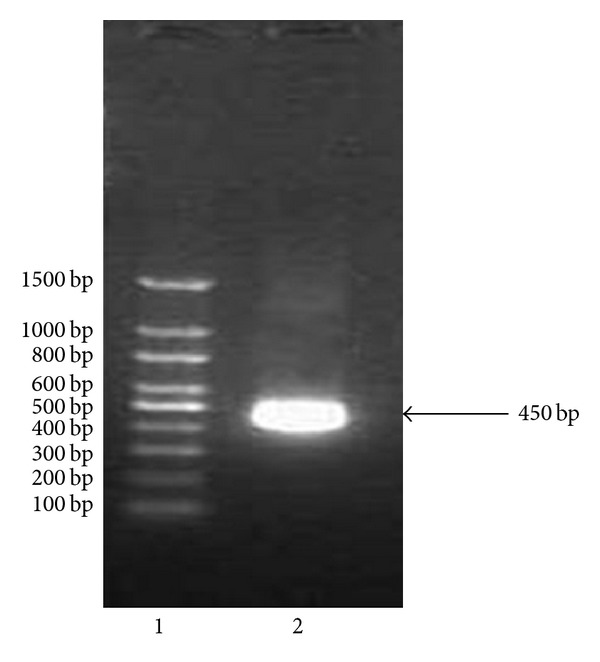
Agarose gel showing the amplification of nucleoside diphosphate kinase gene. Lane 1 is 100 bp plus DNA ladder, and lane 2 is amplified Ndk gene.

**Figure 2:**
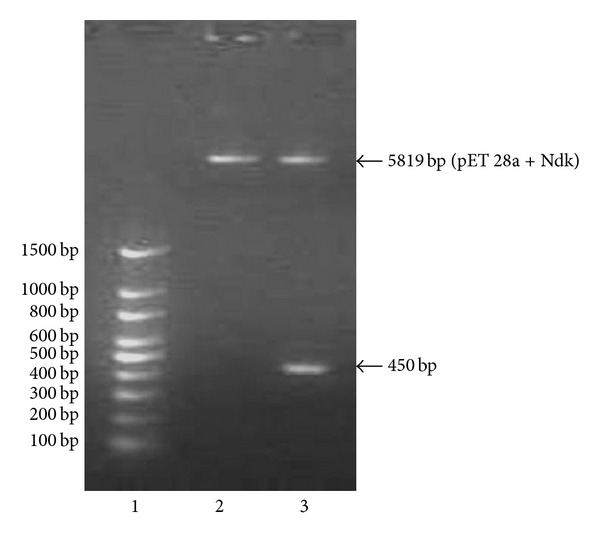
Agarose gel showing confirmation of insert in pET 28a. lane 1 is 100 bp DNA ladder, lane 2 is uncut pET 28a-Ndk construct and lane 3 is restriction digestion of pET 28a-Ndk gene.

**Figure 3:**
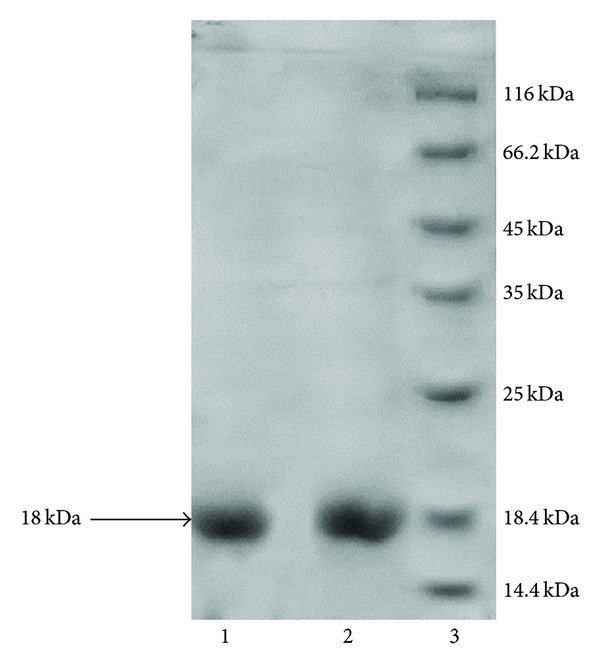
SDS-PAGE showing the purity of protein: lane 1 and 2 is purified Ndk and lane 3 is protein molecular weight marker.

 Specific primers were designed to amplify Ndk from the *Acinetobacter baumannii* (Figure 1). The amplified *Ndk* gene was subcloned in pET 28a vector. The integrity of the recombinant vector pET28a-Ndk was confirmed by double digestion using *NdeI* and *XhoI* restriction enzymes (Figure 2). Identity and orientation of Ndk in the construct was confirmed by sequencing the recombinant vector. Cloned Ndk gene sequence showed 100% homology with the reference sequences. *E. coli* Bl-21(DE3) cells harbouring pET28a-Ndk plasmid were cultured at 37°C in the presence and absence of an inducer IPTG. Induction of the cells with IPTG (0.4 mmol/L) at 37°C for 4 h was found to be optimal to achieve high-level expression of Ndk. Both supernatant and the pellet of cell lysates were tested for the presence of recombinant proteins, and the majority of the expressed protein was detected in supernatant. Recombinant protein was carefully purified with Ni-NTA affinity chromatography (Figure 3). The output of Ndk was approximately 50% of the total bacterial proteins, and the highest detectable level of purified Ndk was up to 3 mg/mL. Further studies in this regard will help in understanding the physiological role of Ndk in these pathogenic bacteria. 

**Figure 4:**
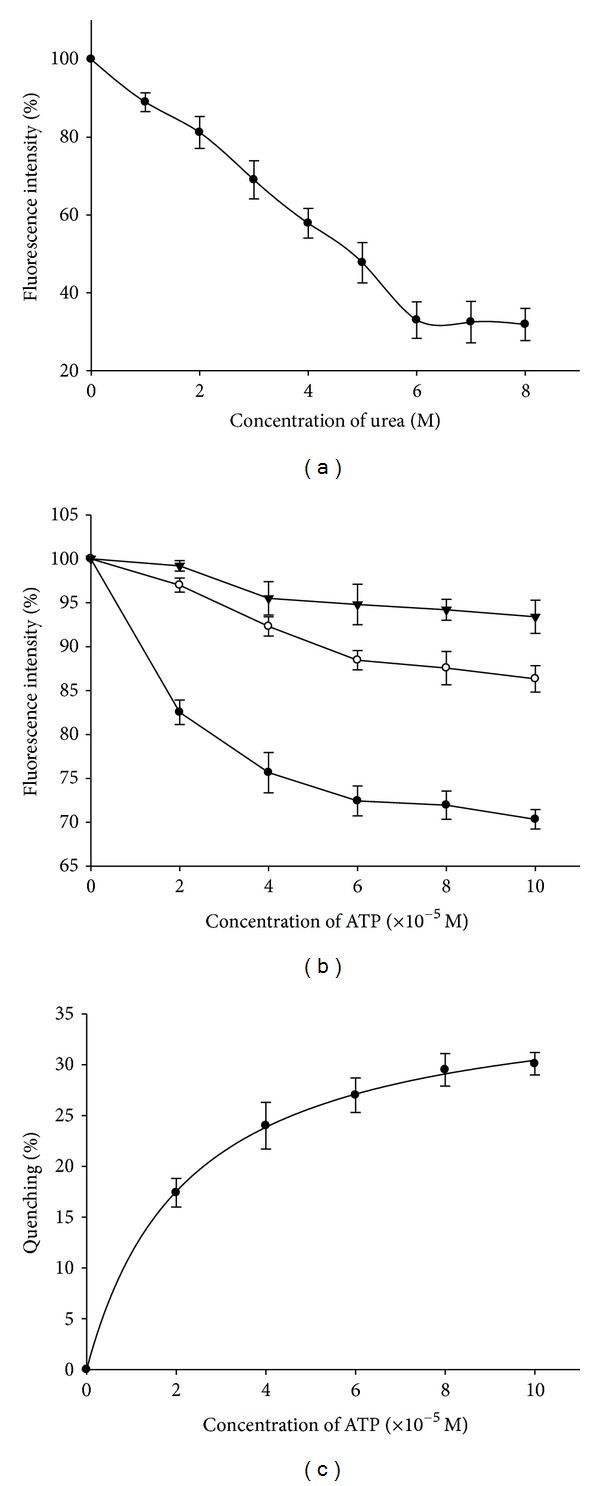
(a) Effect of increasing urea concentrations on fluorescence intensity of Ndk. (b) Effect of increasing concentrations of ATP on fluorescence intensity of Ndk at 3 different pH conditions: acidic (*▼*), neutral (●) and basic (■). (c) The binding curve of ATP with Ndk. The quenching *Q* = (*F*_*o*_ − *F*)/*F*_*o*_ in percent was plotted against increasing ATP concentrations.

 Further characterization studies of this enzyme in terms of stability, pH, and substrate concentrations were carried out by exploiting the fluorescence properties of the enzyme. The fluorescence spectra were recorded in the presence of increasing concentrations of urea (0 M–8 M). Fluorescence intensities in percent were plotted with increasing urea concentrations (Figure 4(a)). The decrease in fluorescence intensity of the protein with increasing urea concentration indicates denaturation of Ndk by urea [17], and complete denaturation was observed after 6 M urea. The error bars on the experimental points were calculated from the average of values that were obtained by repeating each experiment five times.

 Binding of the substrate ATP with the enzyme was confirmed by the quenching of fluorescence intensity with increasing concentrations of ATP [18, 19]. The spectra for the enzyme with increasing ATP concentrations were recorded at 3 different pH conditions. The fluorescence intensities in percent were plotted with increasing ATP concentrations at different pH conditions (Figure 4(b)). Maximum quenching was observed at neutral pH indicating maximum binding of ATP with Ndk at neutral pH. The observed fluorescence data at neutral pH were further used for estimating the resultant fluorescence quenching defined as *Q* = (*F*_*o*_ − *F*)/*F*_*o*_. Plot of *Q* in percent against ATP concentration was prepared (Figure 4(c)). The *R* ^2^ values which indicate the goodness of the fit of the curve were obtained using SigmaPlot 8.0 [20] and were 0.99. The error bars on the experimental points were calculated from the average of values obtained by repeating each experiment five times.
